# Antibody Diversity in Cancer: Translational Implications and Beyond

**DOI:** 10.3390/vaccines10081165

**Published:** 2022-07-22

**Authors:** Raghuram Reddy, Joel Mintz, Roei Golan, Fakiha Firdaus, Roxana Ponce, Derek Van Booven, Aysswarya Manoharan, Isabelle Issa, Bonnie B. Blomberg, Himanshu Arora

**Affiliations:** 1 Desai Sethi Urology Institute, Miller School of Medicine, University of Miami, Miami, FL 33136, USA; rxr954@miami.edu (R.R.); fxf236@miami.edu (F.F.); axm735@med.miami.edu (A.M.); 2 Herbert Wertheim College of Medicine, Florida International University, Miami, FL 33199, USA; 3 Dr. Kiran C. Patel College of Allopathic Medicine, Nova Southeastern University, Davie, FL 33328, USA; jm4719@mynsu.nova.edu; 4 College of Medicine, Florida State University, Tallahassee FL 32304, USA; rg15c@med.fsu.edu; 5 Department of Biology, Florida International University, Miami, FL 33199, USA; rponc016@fiu.edu; 6 John P. Hussman Institute for Human Genomics, Miller School of Medicine, University of Miami, Miami, FL 33143, USA; dvanbooven@med.miami.edu (D.V.B.); ici2@miami.edu (I.I.); 7 Department of Microbiology and Immunology, Miller School of Medicine, University of Miami, Miami, FL 33136, USA; bblomber@med.miami.edu; 8 Sylvester Comprehensive Cancer Center, Miller School of Medicine, University of Miami, Miami, FL 33136, USA; 9 The Interdisciplinary Stem Cell Institute, Miller School of Medicine, University of Miami, Miami, FL 33136, USA

**Keywords:** antibody, immunotherapy, cancer, B cell, T cell, macrophage, monoclonal antibodies, vaccines

## Abstract

Patients with cancer tend to develop antibodies to autologous proteins. This phenomenon has been observed across multiple cancer types, including bladder, lung, colon, prostate, and melanoma. These antibodies potentially arise due to induced inflammation or an increase in self-antigens. Studies focusing on antibody diversity are particularly attractive for their diagnostic value considering antibodies are present at an early diseased stage, serum samples are relatively easy to obtain, and the prevalence of antibodies is high even when the target antigen is minimally expressed. Conversely, the surveillance of serum proteins in cancer patients is relatively challenging because they often show variability in expression and are less abundant. Moreover, an antibody’s presence is also useful as it suggests the relative immunogenicity of a given antigen. For these reasons, profiling antibodies’ responses is actively considered to detect the spread of antigens following immunotherapy. The current review focuses on expanding the knowledge of antibodies and their diversity, and the impact of antibody diversity on cancer regression and progression.

## 1. Introduction

**Figure 1:**
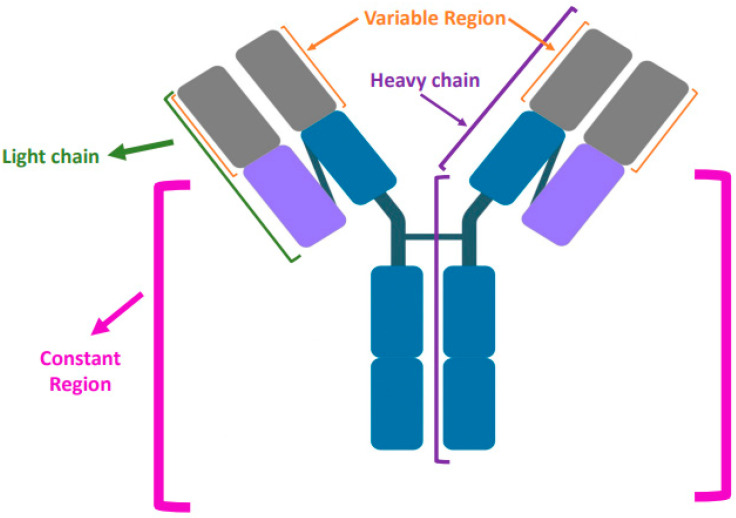
Antibodies have a constant domain, variable domain, heavy chains, and light chains.

Immunoglobulins (Ig), commonly known as antibodies, are Y-shaped proteins used by the immune system to identify and neutralize foreign entities, such as pathogenic bacteria and viruses. All antibodies are composed of either single or repeating units of immunoglobulins, which are composed of two heavy chains and two light chains (Figure 1). In humans, antibodies are classified based on the heavy chain constant regions into five isotypes: IgG, IgM, IgA, IgD, and IgE [1].

Antibodies (Ab) are produced by a subtype of white blood cells called a B cell. They are encoded by unique lymphocyte genes formed during the development of the immune system, resulting from a site-specific recombination between different segments of an immunoglobulin [2]. Genes encode the heavy and light chains that reorganize in B lymphocytes upon activation, which undergo permanent genetic rearrangement of their genes [2]. There are three gene segments of interest on heavy chains, termed the variable (V), diversity (D), and joining (J) sites [3]. 

Every kind of antibody chain has a distinct set of gene segments and exons, wherefrom a polypeptide is formulated [4]. During B cell development, site-specific recombination forms the entire genes for the synthesis of each of the two antibody chains [4]. Moreover, such rearrangements can modify the locations of the enhancers and silencers that affect the promoter, activating transcription [4]. Therefore, complete antibody chains can only be created subsequently after DNA rearrangement. Antigen-binding sites are diverse due to this process of linking gene segments together [4,5]. 

Antibodies, once produced and secreted into circulation, are responsible for different physiological functions. Antibodies have the function of selective antigen recognition and are a main component of the humoral immune response to foreign antigens [6]. This is mediated by the selection of somatic unique antibodies via antigen and T cell help, which contribute to the development of long-term memory B cells [7]. These antibodies function as pathogen neutralizers; they mediate phagocytosis carried out by macrophages, neutrophils, and dendritic cells and realize opsonization and agglutination as mechanisms to defend the body from pathogens and toxins [2]. V(D)J recombination or class switch recombination can occur in developing B cells using DNA nucleases [7]. All mechanisms of antibody diversity, such as the heavy chain rearrangement between D and J gene segments, rearrangement of several hundred V genes with the rearranged D-J segments (VDJ), light chain rearrangement of numerous V and J segments, junctional diversity, nucleotide additions, and combinatorial joining amongst the rearranged H (VDJ) and L (VJ) chains, have a unique function, which increases the possibility for new antibodies to attack any invading pathogens [8].

Antibody diversity is the state in which different antibodies exist, and affinity maturation is the physiological process by which antibodies become increasingly selective for the epitope encountered as a result of somatic hypermutation [9,10]. Generally, antibody diversity stems from a fixed number of genes that create a selection of antibodies, which can select for an essentially limitless number of potential antigens [9]. It has been previously reported that patients with cancer develop antibodies to autologous proteins [11]. These antibodies may arise due to the overexpression of self-antigens, inflammation, or tumor cell lysis. To understand the usefulness of antibody diversity in cancer, the underlying mechanisms need to be comprehended.

On another topic, antibody resistance has become a significant obstacle to the therapeutic use of monoclonal antibodies in cancer treatment [12]. Both tumor-associated and host-related factors have so far contributed to developing resistance against monoclonal antibody therapies [12]. Although current immune-monitoring methods are not yet sufficiently standardized to adequately evaluate immunocompetence routinely, pretherapeutic evaluation is likely to be useful in the future to determine which patients are most at risk of benefiting from or failing Ab therapy [13]. This review focuses on expanding our understanding of antibodies and their diversity, the applications of this understanding in developing biomarkers for early cancer detection, and current advancements.

## 2. Antibody Diversity and Cancer

The immune system has various mechanisms to adapt and fight foreign pathogens. Throughout cancer progression, B cells and antibodies manifest and react through different mechanisms depending on the type and stage of cancer [14]. B cells can disrupt cancer proliferation in cancer types such as breast, epithelial ovarian, melanoma, non-small cell, and renal cell carcinoma through the production of antibodies directed towards unique cancer antigens, promoting destruction by NK cells, phagocytosis by macrophages, and priming CD4+ T cells [15,16]. In tertiary lymphoid structures, B cells associate with T cells within tumors and activate CD4+ T cells to support the B cells in promoting anti-tumor immunity in a variety of ways [17]. In most cancer types, the infiltration of B cells is associated with a good prognosis; however, only some studies argue for a positive prognostic value of B cells [14,18,19,20,21,22,23]. For example, B cells are also reported to promote cancer proliferation by activating myeloid-derived suppressor cells, and the production of autoantibodies, tumor growth factors, and regulatory B cells, which directly and indirectly suppress Th1 and T cell responses [15,24,25]. Studies have shown that infiltrating conventional or B2 cells produce lymphotoxin, which is essential for castration-resistant prostate cancer progression [26]. A study showed that tumor implantation in mice with lymphotoxin deficiencies in B cells caused a significant delay in tumor growth [26]. In addition, tumor-associated B cells expressing STAT3 are reported to produce vascular endothelial growth factor in the tumor site, inducing angiogenesis and further tumor progression [27]. 

One of the mechanisms by which the immune system detects a tumor is through cancer cell lysis following necrosis [28]. Cellular lysis in tumor cells can cause the dispersion of intracellular content in the bloodstream, introducing unique cancer antigens to the immune system [28]. For example, in patients with malignant prostate cancer, a disruption of the barriers between the prostate glandular lumen and its capillaries results in the release of prostate-specific antigen (PSA) into the bloodstream and the subsequent production of anti-PSA antibodies by the immune system [28]. In general, while antibodies against tumor antigens have frequently been found in the serum of cancer patients, the role of humoral immune responses against cancer is yet to be fully understood [29].

As with pathogens, cancer cells can induce a humoral response through their expression of immunogenic neoantigens. Tumor neoantigens may be recognized by the body as foreign and potentially activate antibody production through T cell-dependent or -independent mechanisms. The increase in carcinogenicity results from the instability of genes and subsequent point mutations, which, eventually, may cause the loss of recognition by the immune system [28]. 

It remains unclear whether autoimmune diseases are associated with an increased risk of cancer. In this context, studies comparing patients with systemic lupus erythematosus (SLE) with a control population suggest that SLE increases the risk of non-Hodgkin lymphoma, lung, liver, and thyroid malignancies and reduces the risk of breast and prostate cancers [30,31]. Similarly, studies suggest that tumor cells can trigger humoral immunity, showing that the appearance of certain autoantibodies is part of a defensive, although sometimes futile, immune response against a developing tumor [30]. In this regard, studies have reported an association between B cell malignancies and the incidence of other cancers [32]. For example, Tao et al. reported elevated rates of subsequent Hodgkin lymphoma, lung cancer, and liver cancer amongst diffuse large B cell lymphoma survivors as compared to the control population [33].

It has been argued that tumor-infiltrating B cells may correlate with advantageous outcomes. For example, some studies reported improved survival and lower relapse rates for ovarian cancer, non-small lung carcinoma, and cervical cancer when CD20+ B cell tumor-infiltrating lymphocytes were present [34,35,36]. Potential pathways underpinning B cell anti-cancer immunity could entail B cell secretion of effector cytokines (i.e., IFNγ) [37]. This secretion, in turn, could contribute to Th1/Th2 polarization of T cells or boost the responses of T cells [37]. Furthermore, the possible defensive effect of B cells in cancer is shown by CpG-activated B cells that utilize TNF-related apoptosis inducing ligand (TRAIL/Apo-2L)-dependent mechanisms to attack tumor cells [38,39]. The existence of this function of mediating anti-cancer cytotoxicity is further strengthened by the induction of an increase in granzyme B levels in B-chronic lymphocytic leukemia cells when treated with IL-21 and CpG oligodeoxynucleotide [39,40]. 

Although B cells display cancer-regressive activity, mainly through their antibody production, they can also contribute to tumor progression [25]. Macrophages are reported to play a suppressive role against tumors by capturing the antigens before they reach the nearby draining lymph nodes to activate B cells [25]. In addition to inducing antibody production, B cells also contribute to the increase in Circulating Immune Complexes (CICs). CICs are created when antibodies are bound to free-floating antigens. These complexes can accumulate in tissues, form a site of inflammation, activate complement pathways, and engage the Fc-gamma receptors (FcγRs) on the surface of leukocytes [41]. Therefore, it is not surprising that high levels of CICs are associated with poor prognosis in some cancer patients, such as in breast, genitourinary, and head and neck cancers [42,43]. CICs can result in chronic inflammation through the activation of myeloid cells via engagement of the Fc receptor [44].

The pro-inflammatory and pro-tumorigenic characteristics of CICs were demonstrated by de Visser et al. on a genetic mouse model of squamous cell carcinoma showing that CICs accumulated in healthy tissue induce chronic inflammation by activating the FcγRs on nearby myeloid cells [45]. The activation of myeloid cells, including mast cells and macrophages, triggers the complement pathway, which amplifies angiogenesis, hyperproliferation, and, ultimately, malignant tumors [46]. In another study by Andreu et al., it was shown that CICs from HPV16 mice, when injected into syngeneic non-transgenic mice, triggered an acute inflammatory response [44]. This IgG-mediated activation of the FcγRs on resident and recruited leukocytes, including mast cells and macrophages, regulated not only lymphocyte recruitment but also leukocyte activation within the tumor microenvironment [44]. On another note, IgG may induce leukocyte activation via the complement pathway, further leading to chronic inflammation. Therefore, complement pathways may not be excluded as a sensor of IC deposition [44]. Another study reported that renal tumor progression is associated with complement activation and that C5a deposition is associated with myeloid-derived suppressor cell recruitment and subsequent cytotoxic T lymphocytes’ suppression [47]. 

It is also believed that the key tumor antigens that promote antibody production can be integrated into immune complexes if they are either surface-level or intracellular proteins that do not have potential antigenic, tumor-derived, Major Histocompatibility Class I (MHC I)-binding peptides [15]. These CICs are incapable of promoting the activation of CD8+ T cells [15]. Nevertheless, these CICs may be able to bind to myeloid cells in tumors and activate the FcγRs of those cells, thereby suppressing myeloid cells and encouraging tumor formation [15]. Therefore, targeting B cells, antibodies, and the FcγR’s pathway as cancer therapy is an interesting field that is currently being studied. While more research is needed in cancer, not all CICs may be beneficial for cancer as patients with autoimmune disease such as rheumatoid arthritis, multiple sclerosis, and systemic lupus erythematous benefit from B cell depletion therapies [44,48].

## 3. Autoantibodies in Cancer Diagnostics

Autoantibodies are antibodies that target self-antigens. Their prevalence has been reported in many diseases, and cancer is no exception [49]. For example, more than 64 autoantibodies for various proteins and cell cycle regulatory components were identified for lung cancer alone [50]. Tumors, such as small cell lung cancer (SCLC), are known to produce paraneoplastic autoantibodies to natural cells and organs, which can have important implications for tumor-associated outcomes and prognosis [51]. On a positive note, the autoantibodies are beneficial as biomarkers in cancer therapy, and this is due to their non-invasiveness, high stability, and ability to engage in the early detection of cancer [52]. Tumor-generated autoantibodies (TABs) are not routinely or widely used in clinical practice as a first line means of detecting cancer [52].

Autoantibodies are also detected in colorectal cancer (CRC), which is the third leading cause of cancer in the United States (US) and highly preventable and treatable if detected as a precancerous polyp on a routine screening or at an early stage before lymphatic involvement [53]. Amongst the 199 autoantibodies detected for CRC, the mean sensitivity (identifies true positives) and specificity (identifies true negatives) is 24.0% and 96.4%, respectively [54]. Autoantibodies to p53, a common protein dysregulated in cancer, displays an almost 100% specificity, but it has an extremely low sensitivity (range: 4–46%) and may also be detected for other cancers, limiting its utility as a biomarker in CRC [50,54]. Meta-analysis studies highlighted a few autoantibodies that display sensitivities of over 50% in detecting early-stage CRC, which include but are not limited to autoantibodies such as those to survinin―SPAG9, MUCSAC, AKAP4, and ANXA [54,55,56,57,58,59]. There are test kits available commercially to detect autoantibody panels in different cancer types, which is considered a good way to increase the sensitivity of the test but negatively lowers the specificity, illustrating an important sensitivity–specificity tradeoff when designing any test where there is some degree of uncertainty [54]. 

Lung cancer is the number one cause of cancer death in the United States [60]. However, despite a relative reduction in mortality of 20% with low dose CT, screening amongst the eligible population remains low, at approximately 4% in 2015, and baseline screening has a high false positive rate, varying across studies from 7.9% to 49.3% [60,61,62]. As with other cancer types, lung cancer also has a place for autoantibodies as a biomarker. In this context, a meta-analysis study tested a panel of seven autoantibodies to p53, CAGE, NY-ESO-1, GBU4-5, SOX2, MAGE A4, and Hu-D amongst 3613 patients in four studies. The results found a sensitivity, specificity, diagnostic accuracy, and area under the curve (AUC) of 47%, 90%, 78.4%, and 0.90, respectively, to any stage of lung cancer [63]. A different analysis found that another autoantibody to HMGB3 was frequently upregulated in the early stages of lung cancer [64]. Furthermore, the sensitivities of HMGB3 for diagnosing lung cancer were improved by over 40% when combined with traditional protein biomarkers such as CEA, CA125, or CYFRA21-1, suggesting that the utilization of autoantibodies to diagnose cancer should be combined with other approaches to improve sensitivity and test characteristics, which can increase both diagnostic utility and reliability [64]. Given the moderative test characteristics of autoantibodies to lung cancer, future prospective studies are needed to evaluate how such autoantibodies may improve the results of lung cancer screening, especially alongside existing lung cancer screening methods such as low dose CT.

Other antibodies may be similarly utilized for the detection of other malignancies. One promising application is that for ovarian cancer. For example, one study identified a three-panel microarray consisting of GNAS, p53, and NPM1, which had a sensitivity, specificity, and accuracy of 51.2%, 86.0%, and 68.6%, respectively [65]. Numerous other autoantibodies have been studied in ovarian cancer with varying sensitivities and specificities, and combining these results with other panels could further improve test characteristics for the diagnosis of ovarian cancer [66]. Similar utility of autoantibodies for cancer diagnosis may also be useful for breast cancer and other malignancies, although the antibodies and associated test characteristics may differ between cancers and autoantibodies [67,68,69,70,71,72]. Unfortunately, one of the major limitations to the widespread implementation of autoantibodies for the detection of cancer is their inability to detect premalignant lesions, as one study of gastric cancer found autoantibody formation in only 5% of all premalignant gastric lesions as compared to 15% of early-stage gastric cancerous lesions [73]. Thus, further work focusing on the use of autoantibodies for the detection of early, premalignant lesions is in process. This may be most beneficial to patients who are at risk of malignant progression and adverse outcomes, as one study showed that an early serum rise in certain autoantibodies is predictive of prolonged survival in patients with metastatic non-SCLC who received salvage therapy [74]. 

## 4. Autoantibodies in Cancer-Associated Paraneoplastic Syndromes

Paraneoplastic syndromes are a collection of rare disorders, marked by an atypical immune response to a benign or malignant tumor called a “neoplasm” [75]. Numerous paraneoplastic syndromes have been identified in connection with a variety of malignancies. The “classic” cancer type to produce numerous paraneoplastic syndromes is SCLC, which has been commonly associated with Cushing’s syndrome and syndrome of inappropriate anti-diuretic hormone amongst others [76]. Generally, the pathophysiology of antibody-driven paraneoplastic syndromes is thought to be led by an immune attack against tumor antigens, which then forms antibodies that attack non-tumor cells and organs. Another classic autoantibody-induced paraneoplastic syndrome is Lambert–Eaton myasthenic syndrome (LEMS), which is caused by autoantibody formation to voltage gated calcium channels in skeletal muscle and is associated with an underlying SCLC malignancy in about 60% of patients [77]. Interestingly, LEMS may be the first sign of malignancy, with some patients being diagnosed with cancer 6 years after the onset of LEMS [77]. Furthermore, autoantibody formation may also occur in the absence of clinical symptoms, with 25% of lung cancer patients reportedly testing positive for LEMS antibodies despite the relative rarity with which the syndrome occurs [77]. SOX1 antibody testing has also shown high specificity and moderate sensitivity to LEMS and SCLC [77]. Nevertheless, autoantibody formation is noted in approximately 1–2% of healthy patients, thus clouding the ability of these antibodies to be successfully used in the diagnosis of cancer [77]. Interestingly, it has been reported that those with LEMS live longer than those without clinical evidence of the syndrome, and that 90% of patients and 40% of all newly diagnosed patients were seropositive for other paraneoplastic antibodies without the presence of syndromes [51]. Other common tumors have also been reported to display paraneoplastic syndromes, which are commonly associated with autoantibodies to neurological components, with a classic association noted between the antibodies to the N-methyl-D-aspartate (NMDA) glutamate receptor and ovarian teratomas, which may cause encephalitis [78]. Paraneoplastic cerebellar degeneration is the most often reported neurological paraneoplastic syndrome, with the underlying antigen under assault by the autoantibody being highly associated with specific types of malignancies [78]. Namely, reversible neuronal (anti-Tr) antibodies, which target Delta/notch epidermal growth factor-related receptor (DNER), are associated strongly with Hodgkin lymphomas, whereas anti-Hu antibodies are associated with SCLC and anti-Yo antibodies with breast and ovarian malignancies [78,79]. However, other neurological paraneoplastic syndromes also exist [78]. Thus, the detection of any paraneoplastic syndrome should prompt a search for an underlying malignancy that is associated with the autoantibody of interest. 

Neurological paraneoplastic syndromes have also been infrequently associated with prostate cancer, as approximately 2.5% of all patients with a tumor-associated neurological paraneoplastic syndrome had an underlying prostate carcinoma [80,81]. Generally, paraneoplastic syndrome was highly associated with anti-Hu antibodies, and this was irrespective of advanced clinical course [81]. The authors found that paraneoplastic syndromes were more prevalent in neuroendocrine prostate tumors relative to their incidence, as one-fourth of recognized paraneoplastic syndromes in prostate cancer occurred in neuroendocrine tumors despite this subtype representing less than 3% of all prostate cancer types [81]. Nevertheless, these results may suffer from publication bias in published studies due to the infrequent nature of these paraneoplastic syndromes [81]. It remains to be determined if the detection of autoantibodies, particularly those to the Hu antigen, can be used as a diagnostic or prognostic biomarker for the treatment of prostate cancer. 

## 5. Designer Antibodies for the Detection of Cancer

A potential novel application of antibody diversity in cancer diagnostics is the use of designer antibodies to detect cancer. Traditional approaches have utilized these antibodies intraoperatively such that the surgeon can better visualize surgical margins with instruments that can detect radioactive or fluorescent signatures, which can reduce postoperative recurrence and improve surgical margins [82]. For example, conjugation of panitumumab, an epidermal growth factor receptor (EGFR) antibody, and other designer antibodies to infrared probes in melanoma cell lines successfully delineated the tumor from normal tissue upon systemic injection [83]. When such antibodies were combined with detective devices, such as in early studies that utilized radioactive iodine-125 conjugated tumor-associated glycoprotein 72 (TAG72), they had localization rates ranging from 70% for colorectal cancer to 100% for prostate cancer [82]. More recent clinical trials using fluorescent anti-CEA antibodies have shown great promise for using similar methodology [84]. Nevertheless, these methods require the cancer to be detected using more traditional methods, which may strictly limit the usage of novel antibodies to early stage, resectable malignancies. 

Other approaches utilize designer antibodies for improved detection via imaging methodologies. For example, using anti-EGFR antibodies and gold-coated nanoparticles improved malignancy detection in lung cancer [85]. Other tests using antibodies in common cancer screening methods such as mammography or colonoscopy have not been well characterized. However, Tn antigen (GalNAcα-O-Ser/Thr)-targeting antibodies may be an ideal starting point for novel cancer detection methodology, although it may be limited by differing specificities of the antibodies to the Tn amino acid backbone [86]. Yet, many studies have approached cancer detection with non-traditional methodology, which suggests the use of novel cancer antibodies need not be limited by existing diagnostic tools. The utilization of salivary autoantibodies to aid in cancer detection has been shown to work in proof-of-concept studies, and similar studies using designer antibodies against targets in the serum, saliva, or urine may be similarly successful [87,88]. Nevertheless, this assumes that the targeted antigen is expressed highly in one of those three targets and has significant enough sensitivity and specificity to yield high-quality diagnostic results. One approach would be to design antibodies specific to readily formed antigens on cancer cells, which would improve their sensitivity and specificity for cancer detection. This process would involve finding the best antigen through antibody-independent means, the subtractive hybridization of cDNA, and transcription/translation to obtain the peptide.

## 6. Future Directions: Advancement in Cancer Therapies

**Figure 2:**
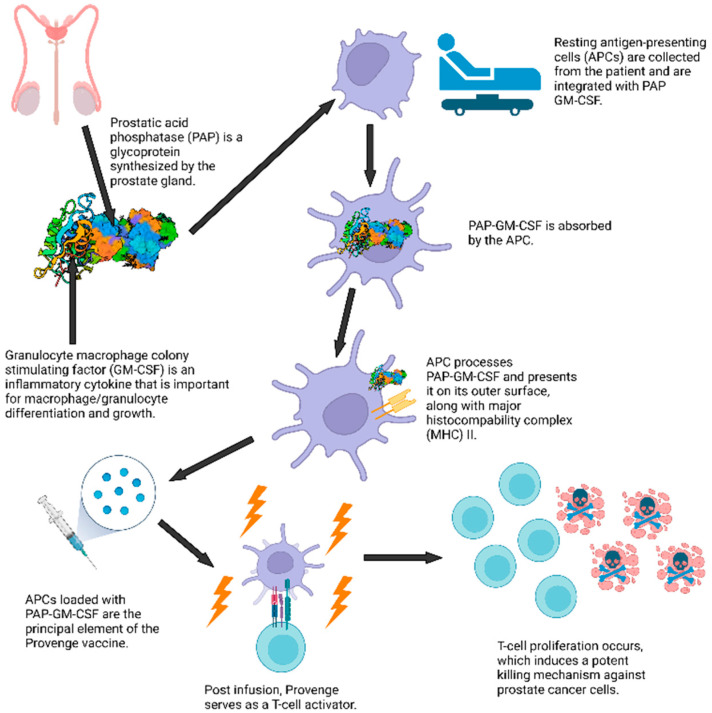
Provenge, the first FDA-approved cancer vaccine, works through utilizing a recipient’s immune cells and prostatic acid phosphatase (PAP) as part of the vaccine to stimulate T cells to attack prostate cancer cells. Created with BioRender.com for this review.

Amongst several treatment options available against different cancer types, immunotherapy, centered on antibody diversity, has become a noteworthy treatment. Immunotherapy refers to drugs, biologicals, vitamins, transplantation, and immunizations that are utilized to trigger a patient’s immune system to discover and attack cancerous cells [89]. For example, one type of immunotherapy for prostate cancer is a cancer vaccine called Sipuleucel-T (Provenge) [90]. This vaccine uses a patient’s own immune cells mixed with prostatic acid phosphatase (PAP), a protein associated with prostate cancer cells, to create a personalized dose (Figure 2) [91,92]. The dose given to the patient enables the cells infused by the vaccine to help the patient’s immune system cells fight prostate cancer [90]. While this vaccine does not stop cancer growth, it has been shown to be statistically significant for survival expansion [91]. Since Provenge was approved, four cancer vaccines for metastatic castration-resistant prostate cancer have been tested in phase III clinical trials: GVAX-Pca, Prostvac (PSA-TRICOM), personalized peptide vaccine (PPV), and DCVAC/Pca [92].

Immunoglobulin replacement therapy is another option, and this treatment is administered to patients with immunodeficiencies such as hypogammaglobulinemia [93]. Patients with these low antibody levels suffer from chronic infections and a low immune response. The basic therapy is derived from immunoglobulins that have been donated from healthy blood plasma [94]. Alternatively, monoclonal antibody therapy has been advanced recently as another therapy option. Monoclonal antibodies are produced in a lab setting that can enhance or mimic the immune system response to cells [95]. These antibodies can be mass produced and programmed to focus on specific targets once the antigen is known (e.g., sialylated MUC1 mucin) much in the same way that the natural immune system would [95,96]. However, this treatment often needs to be administered as early as possible in a patient’s disease progression to have maximum efficacy [95]. More work is needed before this becomes a mainstream therapy to a broad category of diseases, but this antibody treatment has shown promise in a public setting recently in both cancer and COVID-19 therapy [97,98,99]. Notably, the monoclonal antibody dostarlimab was recently used in a Phase II study involving 12 patients with mismatch repair-deficient stage II or III rectal adenocarcinoma, and the results remarkably showed a complete clinical response for all patients [100]. 

**Figure 3:**
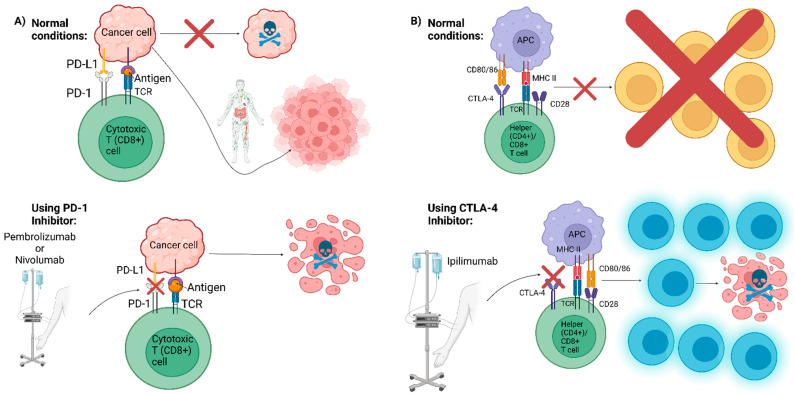
Immune checkpoint therapy. Created with BioRender.com for this review. (**A**) Cytotoxic (CD8+) T cells possess a vital anti-proliferative function. However, cancer cells utilize Program Death-1 Ligand (PD-L1) to deceive the immune system into thinking they are healthy cells, causing the CD8+ cells to become anergic. Pembrolizumab and Nivolumab work by blocking PD-1 interaction with PD-L1. (**B**) When CD28 (co-stimulatory signal) on a helper (CD4+) T cell or CD8+ T cell binds to CD80/86 on an antigen-presenting cell (APC), it activates naïve T cells. However, CTLA-4 competes against CD28 for CD80/86 binding. The binding of CTLA-4 to CD80/86 inhibits the activation of naïve T cells. Ipilimumab inhibits CTLA-4, relieving immunosuppression.

Another type of advanced therapy option against cancer is immune checkpoint therapy. All cells have an immune checkpoint molecule that can act as a signal to the T cell to protect it from an immune response [101]. However, cancer cells can mimic this protection by dysregulating these immune checkpoints [102]. There are two well-known checkpoint receptors that have been well characterized in cytotoxic T-lymphocyte-associated antigen 4 (CTLA-4) and programmed cell death protein 1 (PD-1) [101]. These two checkpoints have been the focus of drugs that work as checkpoint inhibitors, blocking these proteins from interacting with other proteins and thus preventing this protection and allowing the T cell to attack the cancer cells [102]. There is more work to be carried out in this field, but the main examples that have been approved by the FDA include ipilimumab, which interacts with CTLA-4, and pembrolizumab and nivolumab, which interact with PD-1 (Figure 3) [101,103].

Cellular immunotherapy, or adoptive cell therapy, is another novel means to boost the immune system by increasing the immune cellular numbers derived from various sources [104]. This category of therapy includes tumor-infiltrating lymphocyte (TIL) cell therapy, engineered T cell receptor (TCR) therapy, chimeric antigen receptor (CAR) therapy, and natural killer (NK) therapy [104]. Each of these therapies are designed to enhance the current immune system response from either normal cells or from the tumor itself [104]. In some of these therapies, T cells have also undergone gene therapy to strengthen their cellular response [105]. While most of these therapies work when a patient has their immune cells extracted, some work has also been conducted on obtaining partially HLA-matched immune cells from allogeneic sources [106,107]. Further work must be carried out in this area on how to minimize antibody-mediated rejection, but a potential goal for this type of therapy would be for individuals with a high immune response to be able to readily donate their cells for patients with a low immune response, thus giving another tool for which a general therapy could be constructed. 

Immunotherapy is not without its flaws and can fail for some cancer patients. Resistance to immunotherapy is, in part, due to genetic alterations. For instance, RET rearrangements and HER2 mutations can contribute to low tumoral PD-L1 expression, thereby decreasing the probability of a response to immunotherapy [108]. Additionally, aberrations in downstream mechanisms such as the AKT/PTEN, WNT/ β-catenin, JAK/STAT, and MAPK signaling pathways can contribute to immunotherapy resistance [109,110]. With regards to the AKT/PTEN signaling pathway, this resistance may be attributed to the suppression of PI3K signaling and the decreased activity of TILs [109]. Other factors contributing to immunotherapy resistance include the scarcity of tumor-infiltrating CD8+ T cells, PD-L1 overexpression, loss/overexpression of PD-1, lack of neoantigens, and upregulation of additional immune checkpoint receptors (i.e., CTLA-4, LAG 3) [110,111,112]. While there is a possibility of immunotherapy resistance, it should be understood that this is only for a subset of cancer patients. Notably, there are promising clinical trials underway that are investigating overcoming immunotherapy resistance, one being NCT05304546, a Phase 2 trial using pembrolizumab, encorafenib, or binimetinib to overcome primary resistance to immunotherapy (singular inhibitor or immune checkpoint blockade) in Stage IV or inoperable stage III metastatic melanoma patients with a BRAF V600 E/K mutation [113,114]. 

## 7. Conclusions

The importance of antibodies and their diversity in cancer and its progression has been discussed for a long time. However, some of the specifics of antibody diversity/specificity as it pertains to cancer have yet to be unraveled. Immunotherapy options have become more researched and advanced in recent years, though the adaptations of these novel therapies are not yet mainstream options for all cancer types in all patients, and naturally, the same therapy will not be optimal for every single patient. Thus, given the extreme heterogenous nature of cancers and cancer patient responses, the next step will be to enhance these tools for physicians to be able to individualize these therapies to give patients the best possible outcomes.
